# Seed Germination Ecology of *Echinochloa glabrescens* and Its Implication for Management in Rice (*Oryza sativa* L.)

**DOI:** 10.1371/journal.pone.0092261

**Published:** 2014-03-18

**Authors:** Jhoana L. Opeña, Bhagirath S. Chauhan, Aurora M. Baltazar

**Affiliations:** 1 Crop and Environmental Sciences Division, International Rice Research Institute, Los Baños, Laguna, Philippines; 2 University of the Philippines Los Baños, Los Baños, Laguna, Philippines; International Rice Research Institute, Philippines

## Abstract

*Echinochloa glabrescens* is a C_4_ grass weed that is very competitive with rice when left uncontrolled. The competitive ability of weeds is intensified in direct-seeded rice production systems. A better understanding is needed of factors affecting weed seed germination, which can be used as a component of integrated weed management in direct-seeded rice. This study was conducted to determine the effects of temperature, light, salt and osmotic stress, burial depth, crop residue, time and depth of flooding, and herbicide application on the emergence, survival, and growth of two populations [Nueva Ecija (NE) and Los Baños (IR)] of *E. glabrescens*. Seeds from both populations germinated at all temperatures. The NE population had a higher germination rate (88%) from light stimulation than did the IR population (34%). The salt concentration and osmotic potential required to inhibit 50% of germination were 313 mM and −0.24 MPa, respectively, for the NE population and 254 mM and −0.33 MPa, respectively, for the IR population. Emergence in the NE population was totally inhibited at 4-cm burial depth in the soil, whereas that of the IR population was inhibited at 8 cm. Compared with zero residue, the addition of 5 t ha^−1^ of rice residue reduced emergence in the NE and IR populations by 38% and 9%, respectively. Early flooding (within 2 days after sowing) at 2-cm depth reduced shoot growth by 50% compared with non-flooded conditions. Pretilachlor applied at 0.075 kg ai ha^−1^ followed by shallow flooding (2-cm depth) reduced seedling emergence by 94−96% compared with the nontreated flooded treatment. Application of postemergence herbicides at 4-leaf stage provided 85−100% control in both populations. Results suggest that integration of different strategies may enable sustainable management of this weed and of weeds with similar germination responses.

## Introduction

The looming water crisis, increasing labor cost, and shortage of labor have led to a shift from transplanted to direct-seeded rice production systems in many Asian countries [1], [2]. In the Philippines, the availability of water for irrigation has been declining since the last decade. Recently, significant yield loss (34−44%) in transplanted rice was reported at Cavite Province in the Philippines and it was mainly due to water scarcity [3]. Rapid adoption of direct-seeded rice has since been observed in the Philippines. In 1979, only 10% of rice farmers at Nueva Ecija Province in the Philippines used direct-seeding. By 1986, however, 27% of farmers who used transplanting as a method of rice establishment forcibly switched to direct-seeded rice in the dry season (broadcast seeding) and farmers who were practicing a combination of transplanting and wet seeding switched entirely to direct-seeded rice [4]. The moisture status of a field, the introduction of more herbicides, and the lack of timely labor supply for transplanting encouraged this shift to direct-seeded rice [5], [6].

Weed infestation is a serious problem in direct-seeded rice because of the absence of standing water, which has a suppressive effect on weed growth during rice emergence and seedling size advantage of rice seedlings over the weeds [1], [7]. Manual weeding is commonly done, but it is very labor-intensive, is becoming expensive, and getting scarce because of the migration of farm labor to urban areas [1], [8]. Herbicide use is becoming popular as an alternative to manual weeding in many countries. But the intensive use of herbicides poses hazards to human health and the environment, causes evolution of herbicide resistance and shifts in weed population, and increases production costs.

*Echinochloa glabrescens* Munro ex Hook. F., *E. crus-galli* (L.) Beauv., and *E. colona* (L.) Link. count among the world's most serious grass weeds [9]. All *Echinochloa* species have a C_4_ photosynthetic pathway and show a great advantage when grown with C_3_ crops such as rice (*Oryza sativa* L.). These weeds look very similar to rice plants at the seedling stage, causing farmers to sometimes unknowingly transplant these weeds onto the field [10]. By the time the weeds can be easily distinguished from the rice plants, crop yield losses have already been incurred [9]–[11]. *E. glabrescen*s, one of the most common species in lowland rice fields [12], is highly competitive with rice. Uncontrolled *E. glabrescens* transplanted with rice reduced yield by 7% and 87% at weed infestation levels of 5% and 50%, respectively [13]. In another study, yield loss of 90% occurred when all rice hills were infested with *E. glabrescens* seedlings [14].

Better understanding is needed of the factors that affect germination. Such understanding will help in the development of an effective cultural management practice for this problematic weed through either inhibiting its germination or encouraging germination when weed seedlings can be easily controlled [1]. Weed seed germination is influenced by various ecological factors such as temperature, light, soil salinity, soil moisture, seed burial depth in the soil through tillage, and the use of rice residue as mulch. Germination response to these factors is species-specific [15] and such information on *E. glabrescens* is limited in literature.

Weed species, such as *Ipomeoa triloba*, can germinate equally in both light and dark [7], while germination in other species, such as *Leptochloa chinensis*, is completely inhibited in the dark [16]. *E. crus-galli* and *E. colona* do not require light for germination, but light can stimulate their germination [10], [11]. Different weed species also have different responses to salt and osmotic stress. Seed germination was reduced by 50% in *E. colona* at 106 mM NaCl [10] and in *Cyperus difformis* at 23 mM [15]. The osmotic potential required for 50% inhibition of germination in *E. colona* was -0.46 MPa [10]. Seed burial experiments for other related *Echinochloa* species revealed lower germination with increase in seed burial depth. Germination in *E. colona* (97%) and *E. crus-galli* (92%) was highest at the soil surface, and no emergence was seen at 6- and 8-cm depths, respectively [10], [11]. The addition of high amounts (4−6 tons (t) ha^−1^) of rice straw as mulch reduced seedling emergence in *E. crus-galli* and *E. colona* [10], [11].

Different weed ecotypes or weed populations may also have differential responses to the factors that affect germination [17]. In a previous study, for example, two populations of *Trianthema portulacastrum* had differential germination responses to light and seed burial depth [17]. In another study, however, germination response to light, osmotic stress, and burial depth was similar between two populations of *Rottboellia cochinchinensis* [18].

Flooding has long been used as an effective means of controlling weeds in rice. Due to shortage in irrigation water, however, flooding as a method of control may not be possible in the future. In addition, a previous study suggests that flooding alone may not be able to effectively control the *Echinochloa* species. One study suggested that, in order to control *E. glabrescens* in lowland rice, shallow flooding (3 cm) must be combined with suitable tillage systems to lessen the need for manual weeding and herbicide application [12]. In another study, emergence in *C. difformis*, *Ludwigia hyssopifolia*, and *E. colona* was suppressed with the application of a preemergence (PRE) herbicide alongside shallow flooding [8]. The use of postemergence (POST) herbicides is also considered effective in controlling weeds in rice, particularly in direct-seeded rice.

The efficacy of herbicides, however, is dependent on the growth stage of the weed [19], [20]. The efficacy of bispyribac-sodium, penoxsulam + cyhalofop-butyl, and fenoxaprop + ethoxysulfuron on *E. crus-galli*, *E. colona*, and *Digitaria ciliaris*, for example, was reduced when the herbicides were applied at 8-leaf stage compared to when these were applied at 4-leaf stage [19].

A study on the seed ecology of *E. glabrescens* will provide information on the factors that affect its germination and growth, which can serve as a basis in the development of cost-effective control measures of this and other weeds with a similar germination requirement. This study was therefore conducted to determine the effects of light, temperature, salt, osmotic stress, burial depth, rice residue, time and depth of flooding, and flooding depth combined with different rates and application timing of PRE and POST herbicides on the emergence and growth of two populations of *E. glabrescens*.

## Materials and Methods

Experiments were conducted from October 2011 to August 2012 at the International Rice Research Institute in Los Baños, Laguna, Philippines. Seeds of mature *E. glabrescens* were harvested in lowland rice fields of Muñoz City, Nueva Ecija (NE) (latitude 15°43′N, longitude 120°54′E), and Los Baños, Laguna (IR) (latitude 14°10′ N, longitude 121°13′ E), both in the Philippines, in March 2011. The distance between the two sites is approximately 175 km. Seeds were bulked, cleaned, and stored in plastic containers at room temperature until use. The authors confirm that no permission was needed to collect the weed seeds, as well as that the field studies did not involve endangered or protected species.

### Germination test procedure

All experiments were conducted using seeds from both NE and IR populations. To avoid fungal contamination, seeds were soaked in sodium hypochlorite (5%) for 5 minutes and rinsed with running tap water for 3−5 minutes prior to the germination test.

Freshly harvested seeds were tested for germination by placing 25 of the *E. glabrescens* seeds in a 9-cm diameter Petri dish lined with two pieces of Whatman no. 1 filter paper (Whatman International Ltd., Maidstone, U.K.) and 5 ml of distilled water. The Petri dishes were sealed with parafilm and placed inside incubators at fluctuating day/night temperatures of 30/20 °C in light/dark conditions. The photoperiod was set at 12 h to coincide with the higher temperature interval. In all germination tests, seeds with a visible protrusion of the radicle were considered to have germinated. The number of germinated seeds was counted at 15 days after sowing (DAS), or until there was no further germination.

### Effect of temperature and light on germination

Germination in the two populations was determined by placing 25 seeds of *E. glabrescens* in 9-cm diameter Petri dishes. These were then incubated in growth chambers under fluctuating day/night temperatures (35/25, 30/20, and 25/15 °C) in both light/dark and dark regimes. These temperature regimes were selected to reflect temperature variations in the Philippines and other areas in the tropics. For the dark treatment, the dishes were wrapped in three layers of aluminum foil to prevent any light penetration.

The number of germinated seeds was counted at 3-day (d) intervals for up to 15 d for those grown in the light/dark regime and only after 15 d for those grown under dark conditions. Seeds that failed to germinate in the dark after 15 d were moved to the light/dark regime with 5 ml of distilled water added. The number of germinated seeds was counted after 15 d more.

### Effect of salt and osmotic stress on germination

Twenty-five seeds of *E. glabrescens* were placed in dishes containing 5 ml solutions of 0, 25, 50, 100, 150, 200, and 250 mM sodium chloride (NaCl, Mallinckrodt Baker inc., Phillipsburg, NJ) to determine the effect of salt on germination. The solutions were prepared by dissolving 0, 1.46, 2.42, 5.84, 8.77, 11.69, and 14.61 g of NaCl per 1 L of distilled water.

The effect of osmotic stress was studied by placing 25 seeds in solutions with osmotic potentials of 0, −0.1, −0.2, −0.4, −0.6, −0.8, and −1.0 MPa, prepared by dissolving polyethylene glycol 8000 (Sigma Aldrich Co., St. Louis, MO) in distilled water [21].

These ranges of NaCl and osmotic potentials reflect salinity and water-stress levels in the tropics. The Petri dishes were placed inside growth chambers under fluctuating day/night temperatures of 30/20 °C to test germination. Seeds that did not germinate at 250 mM (highest salt concentration) and −1.0 MPa (highest water-stress condition) were rinsed with running water for 5 minutes and placed in the incubator again after adding 5 ml of distilled water. The number of germinated seeds was counted after 15 d.

### Effect of burial depth on seedling emergence

To determine the effect of burial depth, a pot experiment was conducted inside a screenhouse (a chamber of size 10 m×20 m, framed with 2-mm iron mesh and covered overhead with a transparent plastic cover to prevent rain damage). The pots (15.0 cm diameter×15.0 cm height) used in the experiment had holes at the bottom; hence, a piece of paper was placed at the bottom of each pot to prevent soil from leaking out.

The soil used in all pot experiments was collected from upland rice fields; had a pH of 6.6; and was composed of 31% sand, 37% silt, and 32% clay. Soil was autoclaved and passed through a 3-mm sieve. Fifty seeds of *E. glabrescens* from each of the two populations (NE and IR) were sown on the soil surface and then covered with the same soil to depths of 0, 0.5, 1, 2, 4, and 8 cm. Pots were irrigated with an overhead sprinkler initially and, later, subirrigated. In all pot experiments, the visibility of the coleoptile on the soil surface indicated emergence.

The number of seeds that emerged was recorded at 3-d intervals until no further emergence was observed, at 24 DAS.

### Effect of rice residue amount on seedling emergence and biomass

Fifty seeds of *E. glabrescens* from each of the the two populations were sown on the soil surface in plastic pots containing finely chopped rice straw (leaves and stems) of the variety NSIC Rc222. Residue was spread on the soil surface at rates equivalent to 0, 1, 2, 4, and 6 t ha^−1^. The pots and soil used in this experiment were the same as those described in the burial depth experiment.

The number of germinated seeds was recorded at 3-d intervals until no further emergence was observed. At 24 DAS, shoots (leaf and stem) were oven-dried at 70 °C for 72 h and biomass was recorded.

### Effect of depth and time of flooding on seedling emergence and biomass

Fifty seeds from the two populations were sown on the soil surface in small plastic trays (8.0 cm×8.0 cm×5.5 cm). Soil that was used in this experiment was the same as that described in the previous experiment. The pots containing the seeds were placed inside larger plastic pots (15 cm diameter×12 cm height) to retain and maintain water. The pots were marked with 0, 2, 4, and 6 cm to indicate flooding depth. Pots were kept at saturated conditions until flooding was introduced, i.e., at 0, 2, 4, and 8 DAS. Pots were kept flooded at the aforementioned depths for 14 d.

The number of seedlings that emerged was counted at 14 d after start of flooding. The shoot (leaf and stem) and root were separated and oven-dried to constant weights at 70 °C for 72 h to measure biomass.

### Effect of flooding depth and pretilachlor rates on seedling emergence and biomass

Fifty seeds from each of the two populations were sown at 0.5-cm depth in small plastic trays (8.0 cm×8.0 cm×5.5 cm) containing the same soil described in the previous experiments. The pots with the seeds were placed inside larger plastic pots (15 cm diameter×12.0 cm height) to retain and maintain water. The pots were placed on benches inside the screenhouse. These pots were sprayed with pretilachlor (Sofit 300 EC, Syngenta, Fort Bonifacio, Taguig City, Philippines) at 75, 150, 255, and 300 g ai ha^−1^ at 2 DAS. Spraying was done with a research track sprayer (DeVries Manufacturing, Hollandale, MN) with 210 L ha^−1^ spray volume delivery and spray pressure of 140 kPa, fitted with flat fan nozzles (Teejet 80015E, Spraying Systems Co., North Ave. at Schmale Rd, Wheaton, IL 60189). Unsprayed plants were included as the control (nontreated) treatment. Pots were kept at saturated conditions until 5 DAS. Afterwards, flooding was introduced at 0-, 2-, and 4-cm depth and kept at these depths until 14 d.

The number of seedlings that emerged was counted at 14 d after start of flooding. Shoots (leaf and stem) and roots were separated and oven-dried at 70 °C for 72 h and biomass was recorded.

### Effect of POST herbicides on seedling survival and growth

Twenty-five seeds from each of the two populations were sown in small plastic trays (8.0 cm×8.0 cm×5.5 cm). The pots were filled with the same soil as described above. Seedlings were thinned to 6 plants per tray at 3 DAS. The POST herbicides were: bispyribac-sodium (Bayer Crop Science, Canlubang City, Laguna, Philippines) at 30 g ai ha^−1^, fenoxaprop + ethoxysulfuron (Bayer Crop Science, Canlubang City, Laguna, Philippines) at 45 g ai ha^−1^, and penoxsulam + cyhalofop-butyl (Dow Agro Sciences, Ayala Alabang, Muntinlupa City, Philippines) at 72 g ai ha^−1^. Herbicides were sprayed at 4-, 6-, and 8-leaf stages using the research track sprayer described above. A nontreated control was included for comparison.

The number of seedlings that survived (at least one green leaf on the plant) the treatments was counted at 14 d after herbicide application. Shoots (leaf and stem) and roots were separated and oven-dried at 70 °C for 72 h and biomass was recorded.

### Statistical analyses

Experiments were arranged in a randomized complete block design with four replications. Each replication was considered as a block and arranged on different shelves or benches in the incubators or screenhouse. All experiments were conducted twice. The data were combined for analysis, as there were no interaction effects of treatment and experiments. Data were analyzed using regression analysis to determine relationships among different temperature regimes, salt and osmotic concentrations, burial depths, and residue amounts. These were best fitted to a functional three-parameter sigmoid model using SigmaPlot 10.0. The model fitted to the fluctuating temperatures was:
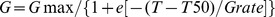
where *G* is the total germination (%) at time *T*, *G_max_* is the maximum germination (%), *T_50_* is the time required for 50% of maximum germination, and *G_rate_* indicates the slope. The model fitted to varying salt and osmotic concentrations was:
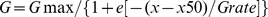
where *G* is the total germination (%) at salt concentration or osmotic potential *x*, *G_max_* is the maximum germination (%), *x_50_* is the salt concentration or osmotic potential required for 50% inhibition of maximum germination, and *G_rate_* indicates the slope. The model fitted to varying burial depth and amount of rice residue was:
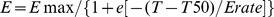
where *E* is the total emergence (%) at time T, *E_max_* is the maximum emergence (%), *T_50_* is the time required for 50% of maximum emergence, and *G_rate_* indicates the slope. An exponential decay curve of the form: 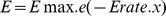
was fitted to seedling emergence (%) obtained at different seed burial depths, where *E* represents cumulative emergence (%) at burial depth *x*, *E_max_* is the maximum emergence, and *E_rate_* indicates the slope. A linear model of the form
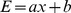
was fitted to seedling emergence (%) obtained at different residue amounts, where *E* represents emergence (%) at residue amount *x*, and a and b are constants. Parameter estimates were compared using the standard error. Treatment means were compared using least significant difference (LSD) at P = 0.05 (GenStat 13^th^ Edition VSN International Ltd., U.K.), when regression analysis was not possible. Analysis was done separately for the two populations.

## Results and Discussion

### Effect of temperature and light

**Figure 1:**
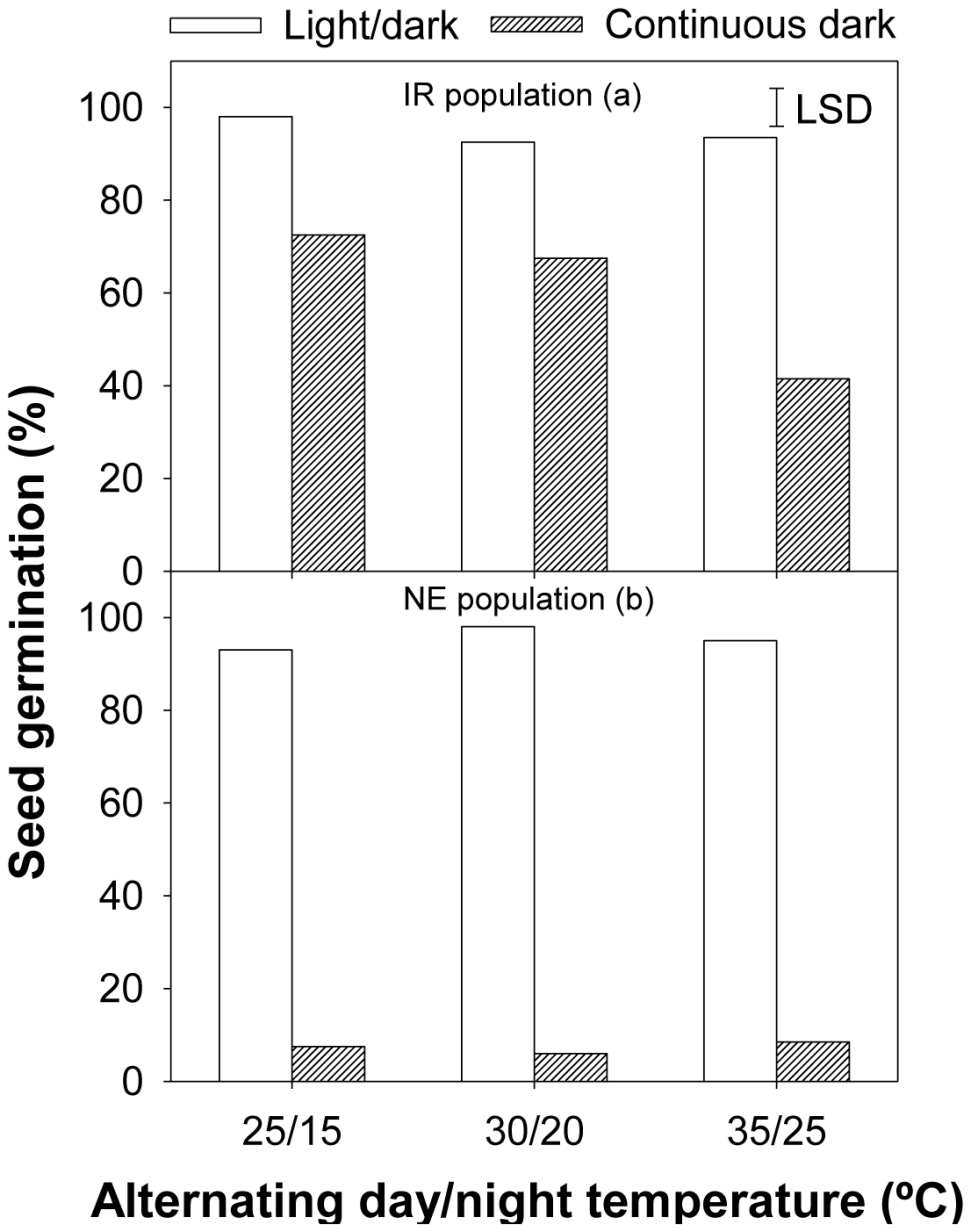
Cumulative germination in two populations (NE and IR) of *Echinochloa glabrescens* incubated at alternating day/night temperatures (25/15, 30/20, and 35/25 °C) and light (light/dark and dark).

Germination of *E. glabrescens* seeds from the IR population was influenced by the interaction between light and temperature (P<0.001) while seeds of the NE population were stimulated by light but not by the alternating day/night temperatures. In the continuous dark conditions, seed germination in the IR population was lower at 35/25 °C (41.5±1.7%) than at 25/15 °C (72.5±5.4%) and 30/20 °C (67.5±2.7%), whereas in the alternating light/dark conditions, germination was similar at all temperatures (Figure 1).

In a previous study, germination in *Eclipta prostrata* at 30/20 °C under light/dark conditions was significantly higher than at 25/15 and 35/25 °C temperature regimes [22]. Germination in *Eleusine indica* was significantly higher in light/dark conditions (68–72%) than in dark conditions (17–25%) at 30/20 and 35/25 °C [23].

Germination of the NE population was more sensitive to dark, as it resulted in an 86–92% reduction in germination. In the IR population, germination decreased by only 25–52%. The differential response of these two populations might be due to the differential conditions—such as water and fertilizer management, nutrient status of the soil, and weather conditions (temperature and sunlight)—during the growth and reproduction of their mother plants.

In our study, we were not able to record these observations at the seed collection areas. A previous study showed contrasting germination responses to heat, salinity, and drought of two populations of *Chenopodium album*, and that the differential germination responses were due to either adaptation via natural selection or maternal effects, or combinations of both [24]. In contrast, a similar germination response was observed between two populations of *R. cochinchinensis* [18].

In the light/dark conditions, seed germination in both populations was similar at all temperatures (Figure 1). The ability of *E. glabrescens* to germinate at all temperatures suggests that this species could emerge throughout the year at low altitudes in tropical countries. Similar results were reported for *E. prostrata*, in which seeds germinated at all test temperatures (i.e., 25/15, 30/20, and 35/25 °C) [22]. Authors suggested a similar speculation regarding germination in *E. prostrata* at all temperatures.

**Figure 2:**
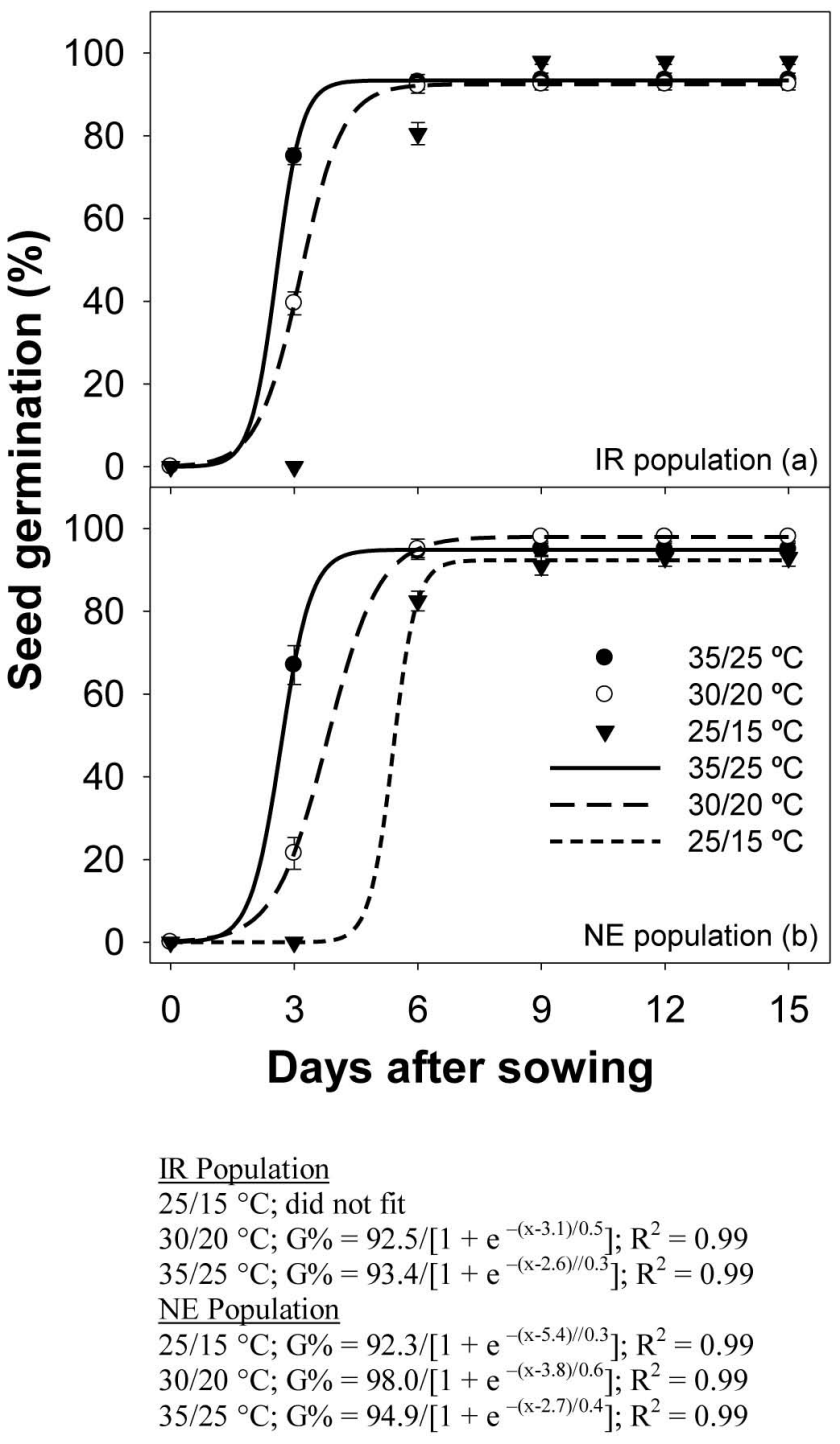
Seed germination in two populations (IR and NE) of *Echinochloa glabrescens* incubated at alternating day/night temperatures (25/15, 30/20, and 35/25 °C) and light (light/dark and dark). The lines represent a three-parameter sigmoid model fitted to the data.

Seeds of both populations, when exposed to light/dark conditions at the lowest temperature regime, took longer to reach 50% germination (*T_50_*) (Figure 2). In the NE population, for example, the time it took for 50% germination (of the maximum germination) at 25/15, 30/20, and 35/25 °C was 5.4±2.8, 3.8±0.01, and 2.7±0.15 d. However, as mentioned earlier, cumulative germination (%) at 15 DAS was similar among test temperatures. Although light was not an absolute requirement for germination, it did stimulate seed germination of the NE and IR populations by 86–92 and 25–52%, respectively.

Weed species vary in germination response to light. Seeds of some species can germinate only with light and others may germinate equally in light or dark conditions. In previous studies, germination in *E. colona* and *E. crus-galli* was stimulated by 39 and 19%, respectively, when exposed to a light/dark regime [10], [11].

When nongerminated seeds from the dark treatment were transferred to light/dark conditions at 30/20 °C, 72–100% of the seeds germinated (data not shown), indicating that the seeds were not adversely affected by incubation in the dark. Similarly, seed germination in *E. prostrata* reached 92–96% when transferred from dark to light/dark conditions [22].

The ecological significance attributed to light response in this species is that amount of light acts as a soil depth indicator, allowing greater germination of seeds on the surface than those buried deeper in soil [25]. The light stimulation germination response of *E. glabrescens* suggests that the species could be a problematic weed in no-till systems, wherein much of the weed seed bank remains on the surface, exposed to light [26]. On the other hand, the exposure of previously buried *E. glabrescens* seeds to light may trigger germination, and that some seeds may still germinate even when buried.

### Effect of salt and osmotic stress

**Figure 3:**
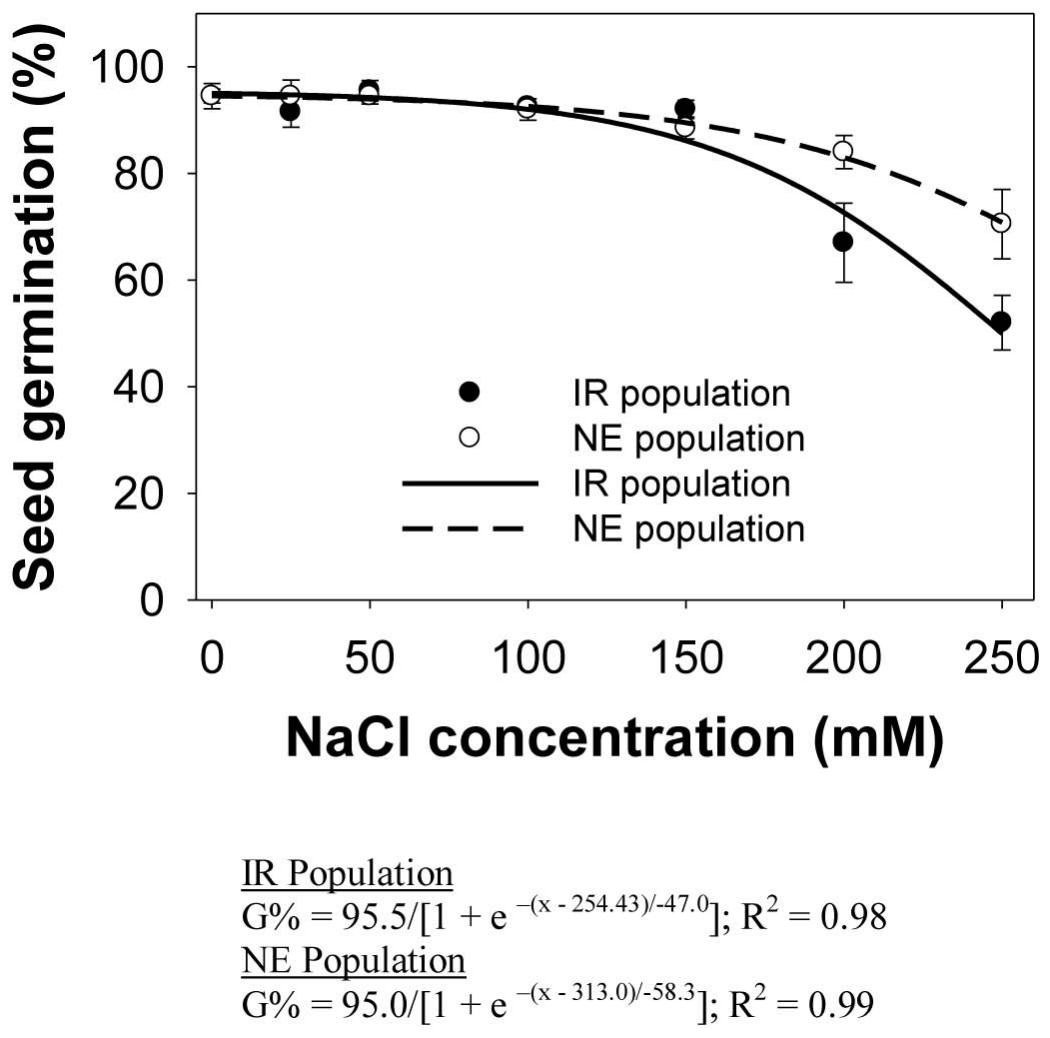
Seed germination in two populations (IR and NE) of *Echinochloa glabrescens*, in response to sodium chloride (NaCl) concentrations when incubated in a growth chamber at 30/20 °C day/night temperature over a 12-h photoperiod for 15 d. The lines represent a three-parameter sigmoid model fitted to the data.

Seed germination in *E. glabrescens* from both populations decreased with increasing salt concentration (Figure 3). Maximum germination (95%) in both populations was observed at 0 mM. Germination in both populations was more than 60% up to a concentration of 200 mM NaCl. Seed germination in the IR and NE populations at 250 mM was still 52% and 71%, respectively. The salt concentration required to inhibit 50% germination in the IR and NE populations was 254 and 313 mM NaCl, respectively. In an earlier study, seed germination in *E. colona* was more than 60% up to a concentration of 50 mM NaCl, and that 106 mM of NaCl was needed to reduce seed germination by 50% [10]. Germination in *E. colona* was completely inhibited at 200 mM NaCl. *L. chinensis*, on the other hand, showed sensitivity to saline stress [16].

When nongerminated seeds after 15 d at the highest tested salinity concentration (250 mM NaCl) were rinsed with distilled water and incubated again at 30/20 °C, germination increased 60–100%, indicating that the seeds were not adversely affected by the saline condition (data not shown). Such a result suggests that seeds in saline conditions may wait until favorable conditions prevail before germination occurs. The longevity of *E. glabrescens* seeds in saline conditions is still unclear, however, and further investigation is needed. Results suggest that *E. glabrescens* will still germinate at high soil salinity, which indicates that the species may be able to grow well in saline areas. Thus, crop cultivation in such areas will be affected not only by salinity but also by competition with this weed species.

**Figure 4:**
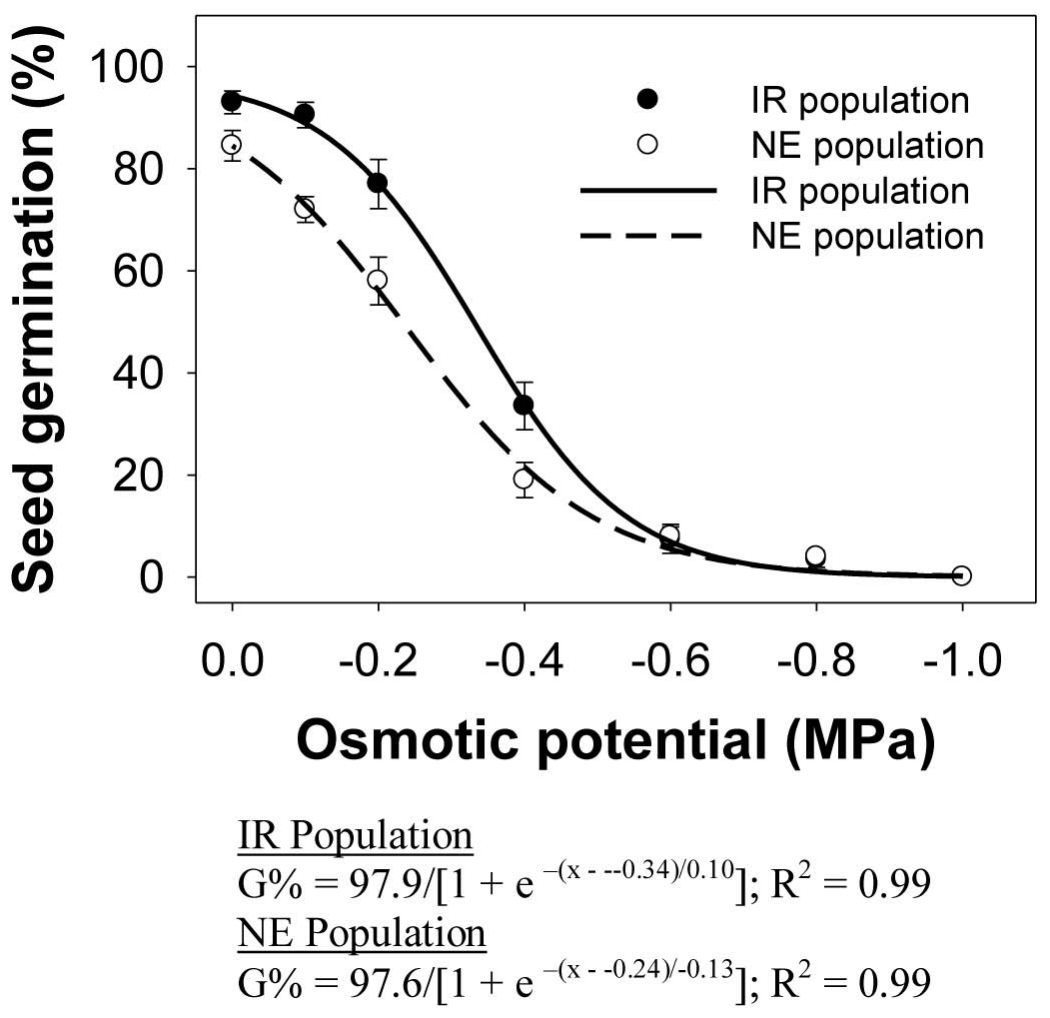
Seed germination in two populations (IR and NE) of *Echinochloa glabrescens*, in response to osmotic potentials (MPa) when incubated in a growth chamber at 30/20 °C day/night temperature over a 12-h photoperiod for 15 d. The lines represent a three-parameter sigmoid model fitted to the data.

Seed germination decreased with reduced osmotic potential in both populations of *E. glabrescens* (Figure 4). Maximum seed germination (98%) in the IR and NE populations was observed at 0 MPa. At osmotic potential of −0.2 MPa, seed germination in the IR and NE populations was 77 and 58%, respectively. An osmotic potential of −0.4 MPa reduced seed germination in the IR and NE populations by 60 and 66%, respectively, compared with the control (i.e., 0 MPa). Some seeds from both populations germinated (4%) at −0.8 MPa, but germination was totally inhibited at −1.0 MPa. The osmotic potential required to inhibit 50% germination in the IR and NE populations was −0.33 and −0.24 mM, respectively.

A similar germination response was observed in *E. colona*, in which germination decreased from 80 to 1% as osmotic potential decreased from 0 to −0.8 MPa and was completely inhibited at osmotic potential of −1.0 MPa [10]. In *E. crus-galli*, increase in osmotic potential allowed increase in germination when seeds were exposed to light [27].

When nongerminated seeds from −1.0 MPa were rinsed and placed in distilled water, seed germinated by 60–80% for the IR population and 40–72% for the NE population. These results indicate that the seeds of *E. glabrescens* were not adversely affected by exposure to low osmotic potentials for up to 15 d. These results also suggest that most of the seeds will germinate in moist conditions, while seeds in dry conditions may wait until moisture conditions are favorable before germination occurs. The ability of *E. glabrescens* seeds to germinate at low osmotic potentials would allow this weed to have a competitive advantage over crops that are susceptible to drought stress. This ability also suggests that germination will be favored immediately after a rainfall, which may coincide with the onset of the wet cropping season in rainfed areas.

### Effect of burial depth

**Figure 5:**
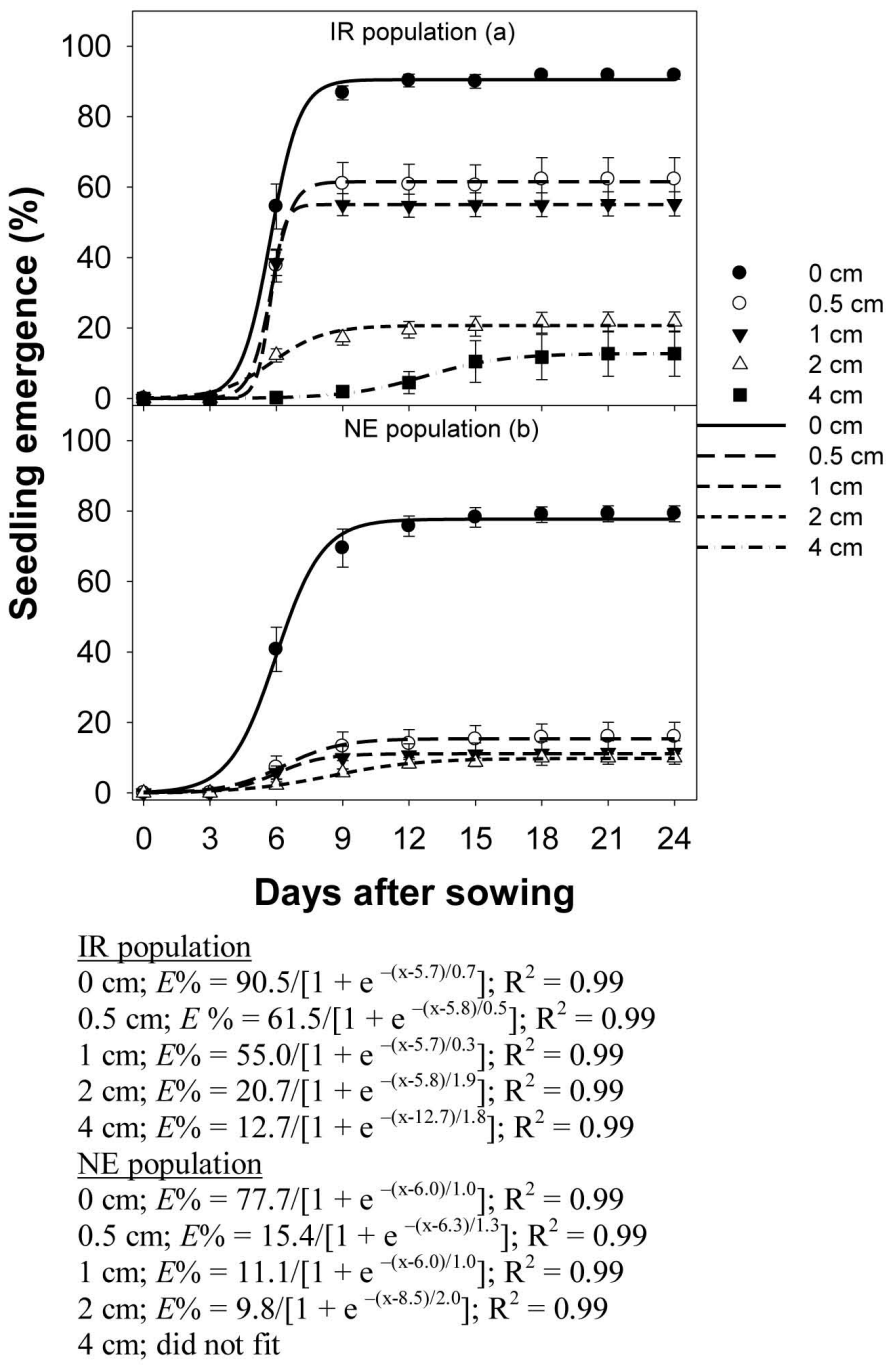
Seedling emergence in two populations (IR and NE) of *Echinochloa glabrescens*, in response to burial depth (cm) when grown in screenhouse conditions for 24 d. The lines represent a three-parameter sigmoid model fitted to the data.

Seedling emergence in both populations of *E. glabrescens* was reduced by seed burial depth (P<0.001). Estimates from the three-parameter sigmoid model indicated that 50% seedling emergence (*T_50_*) was achieved at 5.7 d in the IR population and 6.0 d in the NE population (Figure 5). Fifty percent of the seeds that were buried from 0.5 to 4 cm for the IR population and from 0.5 to 2 cm for the NE population emerged at the same time as seeds that were sown on the soil surface.

On the other hand, the time needed for 50% emergence was delayed by 13 d at 4-cm depth in the IR population, and by 9 d at 2-cm depth in the NE population, compared with seeds sown on the surface.

**Figure 6:**
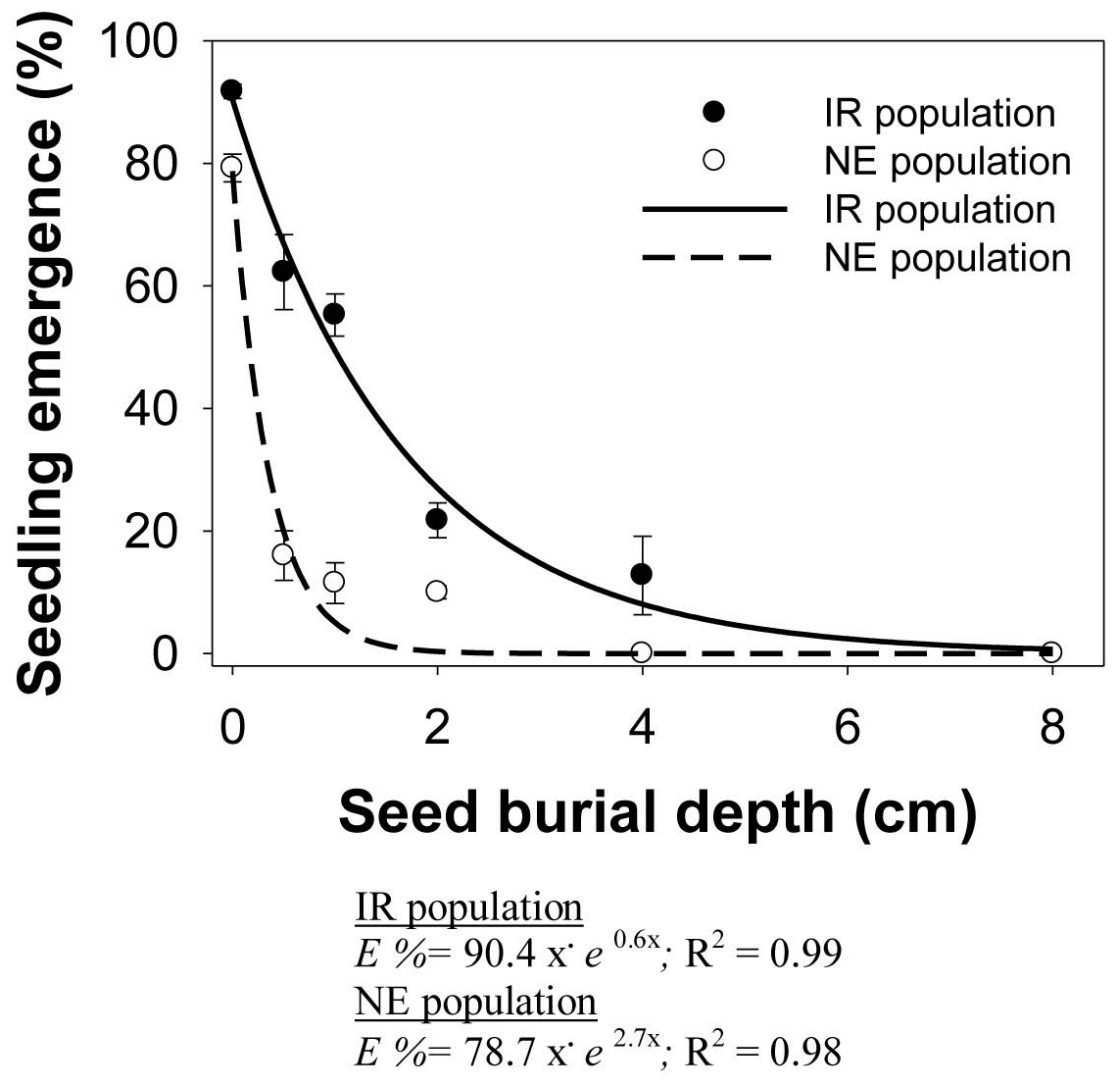
Cumulative seedling emergence in two populations (IR and NE) of *Echinochloa glabrescens*, in response to burial depth (cm) when grown in screenhouse conditions after 24 d. The lines represent an exponential curve fitted to the data.

In both populations, the cumulative seedling emergence at 24 DAS declined with increasing burial depth (Figure 6). The maximum emergence (91 and 79% for the IR and NE populations, respectively) was observed at the soil surface. Seedling emergence in the NE population declined more rapidly with increasing burial depth, compared with that in the IR population. At 0.5-cm burial depth, seedling emergence decreased by 63% in the NE population and 30% in the IR population. No emergence was observed at 4-cm depth in the NE population and at 8-cm depth in the IR population.

The slopes (*E_rate_*) were not similar (0.6 and 2.8) between the IR and NE populations, suggesting that the NE population was more affected by increasing burial depth than was the IR population.

Greater germination on the soil surface in *E. glabrescens* is consistent with stimulation of germination by light. Reduced emergence with increase in burial depth in *E. glabrescens* could be due to the absence of light to signal germination or a limitation on soil gas diffusion, or both [28]. Seeds buried more than 2 mm below the soil surface receive less than 1% of incident light [29]. Another possible reason for reduced emergence with increasing depth could be physical limitations of the seedling, i.e., insufficient seed reserves to enable it to reach the soil surface [18]. The seed mass of *E. glabrescens* was 3.3 mg seed^−1^. Larger seeds with more carbohydrate reserves are more able to emerge from greater burial depths compared with seeds that have lower reserves [30].

It has been reported that the most number of weed species germinated when seeds were placed on the soil surface, although burial depth response varied with the species [15]. Emergence in *Cyperus iria*, *C. difformis, Fimbristylis miliacea*, and *L. chinensis* at depths greater than 0.5 cm was completely inhibited [15]. Emergence in *Digitaria longiflora*, *Heliotropium indicum*, and *Portulaca oleracea* was completely inhibited at 2-cm burial depth [15]. Emergence in *E. colona* decreased by 70–88% at 0.5-cm burial depth compared with that among seeds sown on the soil surface [10], while 11–21% of *E. indica* seedlings emerged at 6-cm burial depth [23].

Stimulation by light and greater emergence on the soil surface in *E. glabrescens* suggests that no-till systems may favor its emergence in the field. This weed species and others with a similar germination response could pose the same problem, particularly in continuous no-till systems.

On the other hand, seeds that remain on the soil surface may be prone to faster desiccation and insect predation [31]. Since emergence is greatly enhanced in these systems, control of *E. glabrescens* before planting is necessary so as to avoid having the crop compete with it. One method is the stale seedbed technique, in which a nonselective herbicide is applied when most of the weeds have emerged before planting. In some areas in the Philippines, farmers practice the rice-maize cropping system, in which glyphosate-resistant maize is grown under a no-till system, thus favoring the use of the broad-spectrum herbicide glyphosate to kill most of the weeds. In order to reduce the build-up of seed banks of this weed species, these tillage systems must undergo rotation. Deep tillage using a moldboard plow that would bury the seeds of *E. glabrescens* below 8 cm in the soil may discourage the weed's emergence. The longevity of buried *E. glabrescens* seeds is still unknown and needs further investigation.

### Effect of residue amount

**Figure 7:**
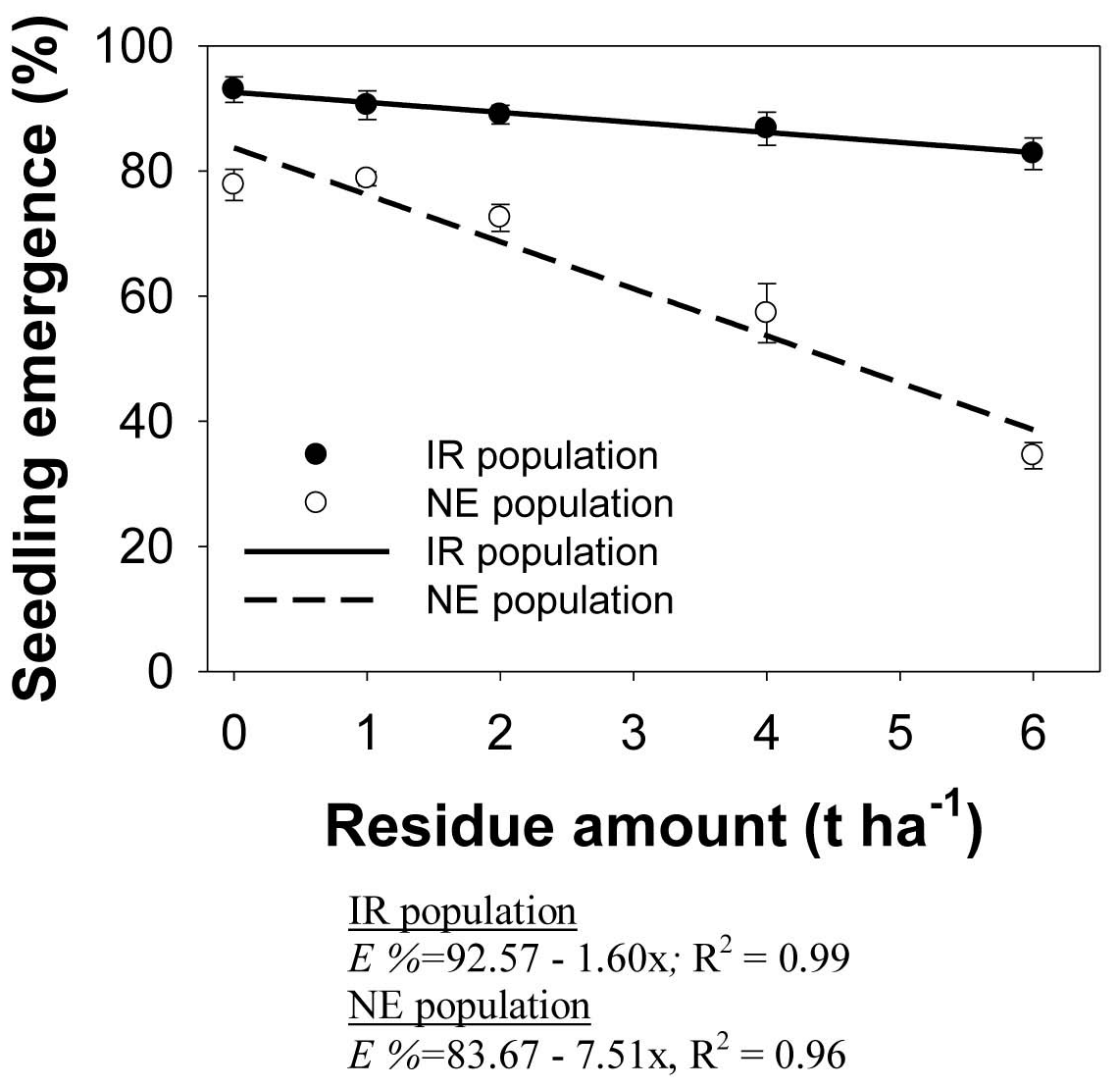
Seedling emergence in two populations (IR and NE) of *Echinochloa glabrescens*, in response to residue amount (t ha^−1^) when grown in screenhouse conditions after 24 d. The lines represent a linear model fitted to the data.

Seedling emergence in the NE (P<0.001) and IR (P<0.03) populations decreased with increasing rates of residues (Figure 7). Maximum seedling emergence in *E. glabrescens* was 93 and 84% in the IR and NE populations, respectively, when no residue was applied. The NE population was more affected than the IR population by increasing amount of residue. Emergence in the NE population increased significantly with the addition of 4 t ha^−1^ residue, although there was still no reduction in emergence in the IR population at this level. The addition of 5 t ha^−1^ of rice residue caused a 55% and 9% reduction in maximum emergence in the NE and IR populations, respectively, compared with when no residue was added.

The difference in response between the two populations is consistent with observations from the seed burial experiment, in which emergence in the NE population was affected more than that in the IR population by increase in burial depth.

Weed suppression by mulch is attributed to various physical and chemical factors. The physical factors include lower soil temperatures, shading, and physical obstruction provided by the mulch itself [32], [33]. No study has yet recorded the rice variety used in our experiments (NSICRc222) as having allelopathic effects. Thus, it is not clear whether the effect of residue on emergence was a result of biochemical or physical factors.

Emergence in many weed species has been reported to decrease with the addition of crop residue. *E. colona* and *E. crus-galli*, for example, were reported to have a marked reduction in seedling emergence with increased amount of residue [10], [11].

The addition of residue at 1–2 and 1–4 t ha^−1^ increased the biomass of *E. glabrescens* seedlings in the NE and IR populations, respectively (Table 1). The addition of rice residue of 4–6 t ha^−1^ in the NE population and 6 t ha^−1^ in the IR population resulted in similar biomass compared with those without rice residue. The increase in biomass with the addition of residue could be a result of higher availability of soil moisture due to better soil-seed contact. Seeds placed directly on the soil surface could have less soil-seed contact, which resulted in slow growth. A similar response was observed in *E. crus-galli*, in which biomass increased with the addition of residue of up to 4 t ha^−1^ [11]. On the other hand, the biomass of *E. prostrata* and *E. indica* decreased as amount of residue increased [22], [23].

**Table 1:** Shoot biomass (g pot^−1^) of two populations (IR and NE) of *E. glabrescens* at different residue amounts (t ha^−1^).

Amount of residue (t ha^−1^)	Shoot biomass (g pot^−1^)	
	*NE population*	*IR population*
0	5.35	4.97
1	7.04	8.32
2	7.13	7.57
4	6.68	8.38
6	3.93	6.59
LSD_0.05_	1.50	2.24

Our results suggest that high amounts of residue were needed to reduce emergence and growth of *E. glabrescens*. These high amounts of residue may not be feasible in the Philippines, as the common practice of majority (95%) of farmers is to burn the rice straw after harvest [34]. In other areas in the Philippines—those where rice hull burning is banned because of air pollution concerns, and in areas with low soil organic matter but which practice organic farming—the addition of high amounts of rice residue mulch may be considered.

### Effect of depth and time of flooding

**Figure 8:**
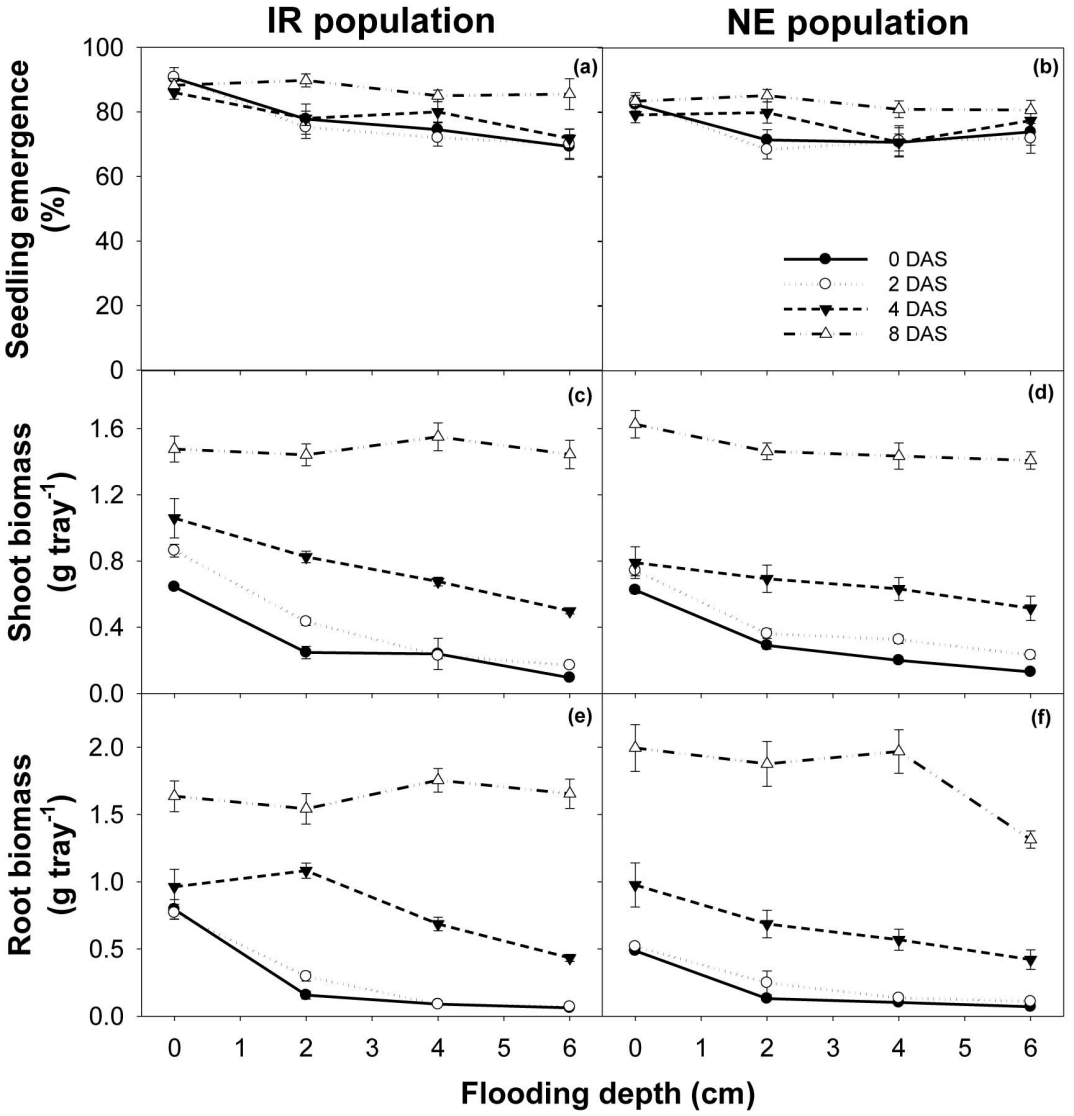
Seedling emergence, shoot biomass (g tray^−1^), and root biomass (g tray^−1^) of two populations (IR and NE) of *Echinochloa glabrescens*, in response to various depth (cm) and time of flooding (0, 2, 4, and 8 days after sowing) when grown in screenhouse conditions. Error bars represent standard error of means.

Seedling emergence and shoot and root biomass in the IR population was influenced (P<0.001) by the interaction between flooding depth and timing. On the other hand, seedling emergence and shoot and root growth in the NE population was influenced (P<0.001) by flooding depth and timing. Regardless of flooding depth, flooding did not kill any seedlings that had emerged (Figures 8a, b). Flooding had a more pronounced effect on shoot and root growth than on seedling emergence in *E. glabrescens* (Figures 8c–f). Timing of flooding, more than depth, affected emergence and growth. On the other hand, depth of flooding became more critical when flooding was delayed.

Although flooding is an important tool for controlling weeds, the response of weeds to flooding varies among species. *L. hyssopifolia* can be easily controlled with flooding [35], but some weeds, such as *Monochoria vaginalis* and *Sphenoclea zeylanica*, are very difficult to control because they are well-adapted to submerged conditions [36], [37]. Similarly, emergence in *E. crus-galli* seedlings can be reduced by flooding at 2 DAS but not at 15 DAS [11].

Reduction in emergence and biomass of weeds in flooded conditions may be a result of several factors that include reduced O_2_ levels; accumulation of CO_2_ and toxic gaseous products of anaerobic decomposition; and the presence of reduced forms of chemical radicals and gases such as methane, nitrogen, nitrous oxides, and sulfides [38]. In addition, the depth-sensing fluctuation in weed seeds might be related to the amplitude of temperature fluctuations. Deeper water could have smaller temperature fluctuations, which might allow less germination [37].

Results showed that, in both populations, flooding at an earlier stage (0–2 DAS) reduced the emergence and shoot and root growth of *E. glabrescens*. Delaying flooding to 4 DAS reduced shoot and root growth but not emergence. At late flooding, reduction occurred only with increased water depth (4–6 cm). Flooding at 8 DAS did not reduce emergence nor growth, regardless of increase in water depth. A similar response was observed in *C. difformis*, *C. iria*, *F. miliacea*, and *L. chinensis*, where flooding depth to as low as 2 cm, compared with 0 cm, greatly reduced emergence when the soil had been flooded continuously for 28 d [15].

These results suggest that, to achieve about 50% reduction in shoot growth, flooding at 2 cm should be introduced not later than 2 DAS. If flooding is delayed up to 4 DAS, then the depth of flooding must be increased. Flooding at 8 DAS did not control emergence and growth in *E. glabrescens*. Seeding depth and flooding reduces germination, survival, and growth in *E. glabrescens* [12]. Thus, in order to control *E. glabrescens* in lowland rice, suitable tillage systems must be combined with shallow flooding (3 cm) to reduce the need for other weed control methods such as hand-weeding and herbicides [12].

In the future, many areas will experience water scarcity and that continuous flooding for weed control will be restricted [39].

The results of our study showed that shallow flooding (2 cm) at 0–2 DAS may significantly reduce emergence and growth in *E. glabrescens* and other similar species. Thus, in areas with limited irrigation water, early flooding at shallow depths rather than late flooding will be a better water management strategy for the control of *E. glabrescens*.

In direct-seeded rice, early flooding could be possible with the use of anaerobic rice cultivars that can emerge despite submerged conditions, and using pre-germinated and/or high-vigor seeds or varieties that can emerge in a short time. By the time the canopy closes, weed growth can be suppressed by shading. However, flooding alone will not completely suppress *E. glabrescens* and still needs to be combined with other weed management strategies. Further investigation of the effect of flooding of different depths and timing under field conditions should be done in the future.

### Effect of flooding depth and pretilachlor rates

Seedling emergence and shoot biomass of both populations and the root biomass of the IR population was influenced (P<0.001) by the interaction between the pretilachlor dose and flooding depth. However, root growth in the NE population was not influenced by the interaction of flooding depth and the pretilachlor dose. It decreased with increase in flooding depth (P<0.003) and increase in the pretilachlor dose (P<0.001).

**Figure 9:**
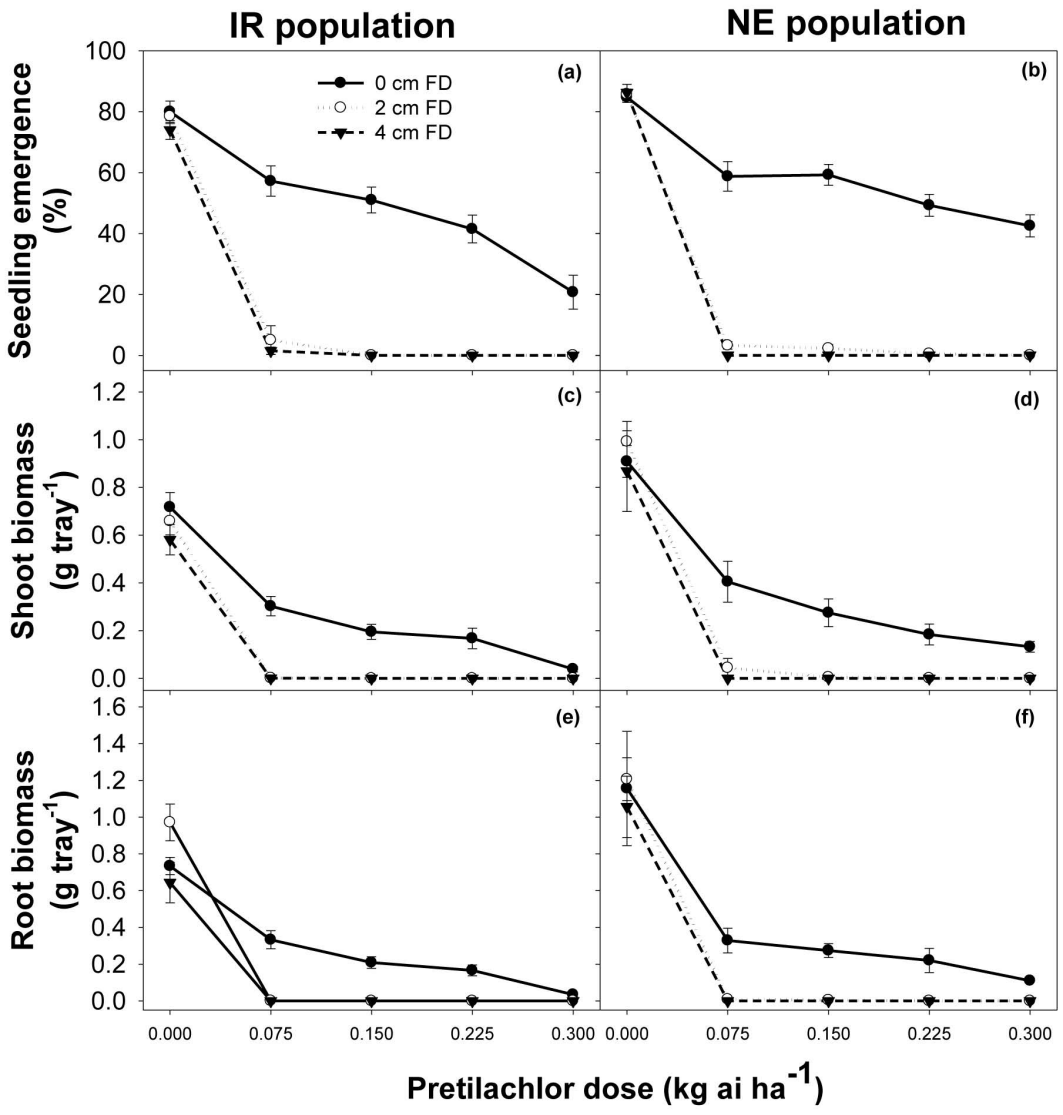
Seedling emergence, shoot biomass (g tray^−1^), and root biomass (g tray^−1^) of two populations (IR and NE) of *Echinochloa glabrescens*, in response to various depth of flooding (cm) and pretilachlor application rates (0, 0.075, 0.150, 0.225, and 0.300 kg ai ha^−1^) when grown in screenhouse conditions. Error bars represent standard error of means.

Flooding alone did not suppress the emergence and growth of *E. glabrescens* (Figure 9). Pretilachlor applied alone also did not completely suppress the emergence of *E. glabrescens* seeds, although it did reduce shoot and root biomass. The IR population showed more sensitivity to increasing pretilachlor dose than the NE population. The emergence and growth of both populations were reduced more by pretilachlor when application was followed by flooding (Figure 9). In flooded conditions, the emergence and growth of both populations were similar at all pretilachlor doses. In saturated conditions, on the other hand, as pretilachlor dose increased, emergence in the IR population and growth in both populations declined.

Results suggest that the application of pretilachlor at 0.075 kg ai ha^−1^ (1/4 of the recommended dose) followed by flooding at shallow depth (2 cm) suppressed the emergence and growth of *E. glabrescens*. This supports earlier observations that pretilachlor combined with flooding controlled *E. glabrescens* in direct-seeded rice [40]. Similar results were also observed in *E. colona*, in which pretilachlor application with subsequent flooding greatly reduced its emergence and growth [8].

Application of pretilachlor alone reduced emergence and growth, but the dose required for such reduction was high (i.e., 0.300 kg ai ha^−1^). Consequently, increasing flooding depth also reduced emergence and growth of *E. glabrescens* but the suppression was not as effective as that with the application of pretilachlor. In *C. difformis*, growth was not affected by flooding alone, but was completely controlled by pretilachlor [8].

In the Philippines, flooding depths in farmers' fields may vary because of poorly leveled fields and limited farm resources [8]. Many rice farmers, now and in the near future, will experience limited water availability that will then restrict their capacity to use continuous flooding as a weed control strategy [39], [41]. Consequently, a shift to direct-seeded rice is anticipated to increase in the future as the availability of water and labor decreases and input costs increase. The results of our study suggest that the use of a lower dose of pretilachlor combined with shallow flooding may provide an effective weed management strategy in direct-seeded rice systems for the control of *E. glabrescens* and weeds with a similar response.

### Effect of POST herbicides

**Figure 10:**
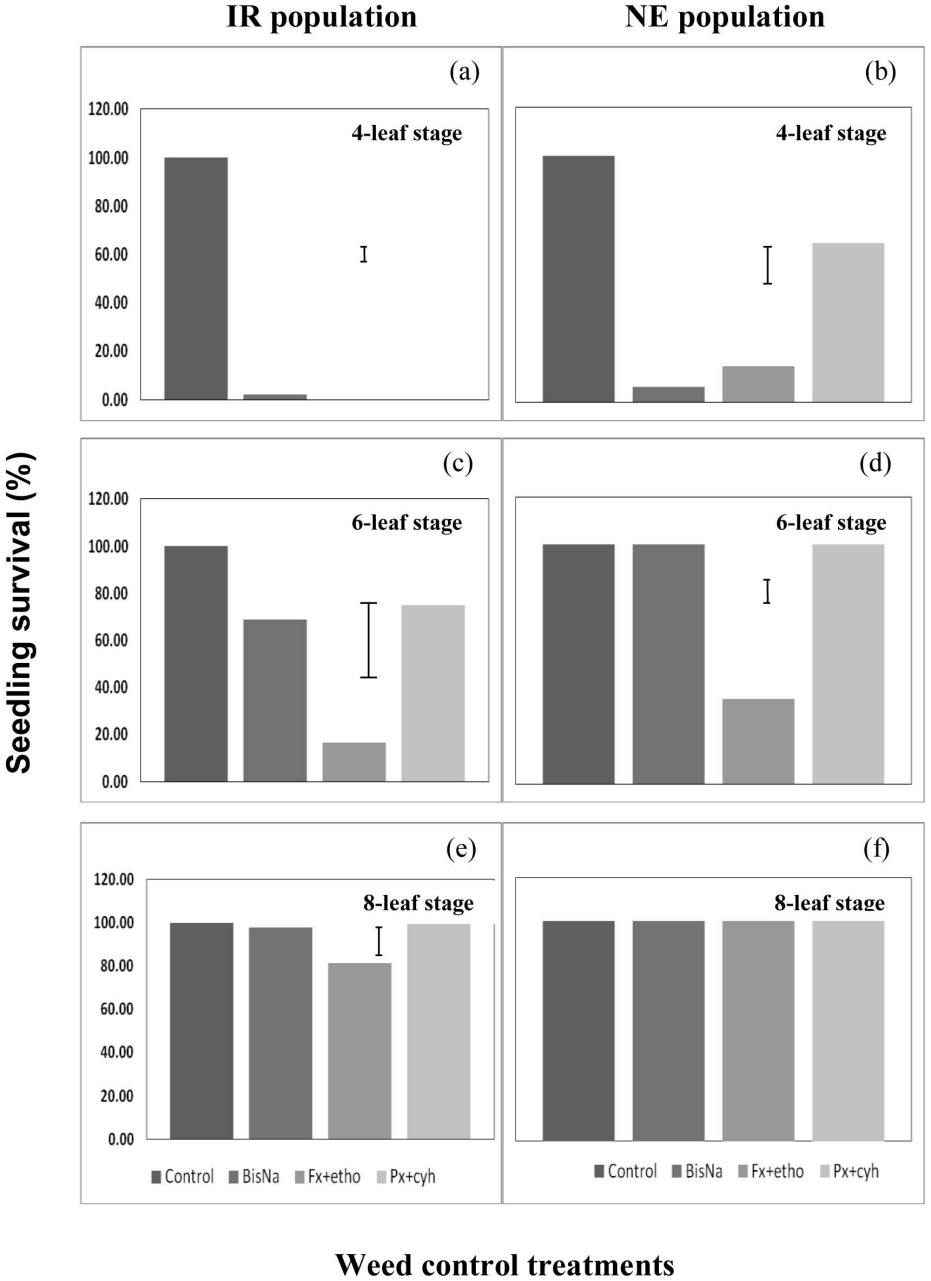
Seedling survival in two populations (IR and NE) of *Echinochloa glabrescens* when treated with postemergence herbicides (BisNa, bispyribac-sodium; Fx + etho, fenoxaprop + ethoxysulfuron; Px + cyh, penoxsulam + cyhalofop) at four-, six-, and eight-leaf stages. Error bars represent least significant difference values at 5% level of significance.

Application of POST herbicides at the 4-leaf stage of *E. glabrescens* reduced seedling survival in both populations. Bispyribac-sodium, fenoxaprop + ethoxysulfuron, and penoxsulam + cyhalofop provided 98–100% reduction in seedling survival in the IR population at the 4-leaf stage (Figure 10a). At the same leaf stage, penoxsulan + cyhalofop provided only a 35% reduction in seedling survival in the NE population, while fenoxaprop + ethoxysulfuron provided an 85–94% reduction (Figure 10b). Efficacy of the POST herbicides on both populations was reduced when applied at the 6-leaf stage (Figure 10c and d), at which only fenoxaprop + ethoxysulfuron reduced seedling survival in both populations. Further delay in herbicide application to the 8-leaf stage no longer reduced seedling survival in the NE population (Figure 10f). At 8-leaf stage, however, fenoxaprop + ethoxysulfuron still caused reduction in seedling survival in the IR population, by 19% (Figure 10e).

The survival of plants treated with POST herbicides increased with age; reduction in surviving seedlings decreased as the application was delayed from the 4-leaf to the 8-leaf stage. Likewise, shoot biomass increased when POST herbicides were applied at later stages. Shoot biomass in both populations was greatly reduced by the application of all POST herbicides at the 4-leaf stage (Table 2), at which bispyribac-sodium, fenoxaprop + ethoxysulfuron, and penoxsulam + cyhalofop were equally effective in reducing shoot biomass in the IR population. However, the commercial mixture of penoxsulam + cyhalofop was less effective compared with bispyribac-sodium and fenoxaprop + ethoxysulfuron in reducing shoot biomass in the NE population at this stage. All herbicides reduced shoot biomass in the IR population by 96–100%. Bispyribac-sodium and fenoxaprop + ethoxysulfuron reduced shoot biomass in the NE population by 67–87%, while penoxsulam + cyhalofop provided only a 41% reduction in shoot biomass.

**Table 2:** Shoot biomass (g tray^−1^) of two populations (IR and NE) of *E. glabrescens* applied with postemergence herbicides (bispyribac-sodium, fenoxaprop + ethoxysulfuron, and penoxsulam + cyhalofop) at four-, six-, and eight-leaf stages.

Weed control treatments	Rate (g ha^−1^)	Shoot biomass (g tray^−1^)		
		Four-leaf	Six-leaf	Eight-leaf
*IR population*				
Nontreated control	0	1.30	1.50	1.48
Bispyribac-sodium	30	0.05	1.20	1.50
Fenoxaprop + ethoxysulfuron	45	0.00	0.78	1.46
Penoxsulam + cyhalofop	72	0.00	1.41	1.49
LSD_0.05_		0.20	0.37	NS
*NE population*				
Nontreated control	0	1.10	1.26	1.76
Bispyribac-sodium	30	0.14	1.34	1.93
Fenoxaprop + ethoxysulfuron	45	0.36	1.18	2.20
Penoxsulam + cyhalofop	72	0.65	1.46	1.76
LSD_0.05_		0.23	NS	NS

Abbreviations: NS  =  not significant.

Efficacy of the POST herbicides was reduced in both populations when applied at the 6- and 8-leaf stages (Table 2). At the 6-leaf stage, fenoxaprop + ethoxysulfuron reduced shoot biomass in the IR population by only 48%. Application of all herbicides at the 6-leaf stage did not reduce shoot biomass in the NE population. Application of these POST herbicides at the 8-leaf stage also did not reduce shoot biomass in both populations (Table 2).

Root biomass of both populations was greatly reduced by all herbicides when applied at the 4-leaf stage (Table 3). Reduction in root biomass when herbicides were applied at this stage was 99–100% and 85–95% in the IR and NE populations, respectively. Application of these herbicides at the 6-leaf stage reduced root biomass in both populations but to a lesser degree compared with when application was made at the 4-leaf stage. All herbicides were equally effective in reducing root biomass in both populations. At the 4-leaf stage, root biomass decreased by 83–87% and 63–76% in the IR and NE populations, respectively. Application of herbicides at the 8-leaf stage reduced root biomass in the IR population by 41–55%, but no reduction was observed in root biomass in the NE population (Table 3).

**Table 3:** Root biomass (g tray^−1^) of two populations (IR and NE) of *E. glabrescens* applied with postemergence herbicides (bispyribac-sodium, fenoxaprop + ethoxysulfuron, and penoxsulam + cyhalofop) at four-, six-, and eight-leaf stages.

Weed control treatments	Rate (g ha^−1^)	Root biomass (g tray^−1^)		
		Four-leaf	Six-leaf	Eight-leaf
*IR population*				
Nontreated control	0	0.80	4.08	1.80
Bispyribac-sodium	30	0.01	0.68	1.00
Fenoxaprop + ethoxysulfuron	45	0.00	0.70	0.82
Penoxsulam + cyhalofop	72	0.00	0.52	1.06
LSD_0.05_		0.06	1.47	0.43
*NE population*				
Nontreated control	0	1.10	2.61	3.41
Bispyribac-sodium	30	0.05	0.97	2.21
Fenoxaprop + ethoxysulfuron	45	0.17	0.62	3.72
Penoxsulam + cyhalofop	72	0.17	0.74	3.39
LSD_0.05_		0.16	0.37	NS

Abbreviations: NS  =  not significant.

Reduction in the root-shoot ratio was observed in both populations and could be a result of the application of POST herbicides at 4- to 6-leaf stages (Table 4). At the 4-leaf stage, bispyribac-sodium, fenoxaprop + ethoxysulfuron, and penoxsulam + cyhalofop all caused similar levels reductions in the root-shoot ratio of the IR population. At the same stage, bispyribac-sodium and penoxsulam + cyhalofop caused similar levels of reduction in the root-shoot ratio of the NE population. There was less reduction in the root-shoot ratio among plants treated with fenoxaprop + ethoxysulfuron (Table 4). When herbicides were applied at the 6-leaf stage, the decrease in the root-shoot ratio was similar in both populations. The decrease in the root-shoot ratio could mean allocation of more biomass to the shoots at the expense of the roots. This may be a survival mechanism of the plant to enable the shoot to recover through photosynthesis. Application of POST herbicides at the 8-leaf stage did not reduce root-shoot ratio in both populations.

**Table 4:** Root-shoot ratio of two populations (IR and NE) of *E. glabrescens* applied with postemergence herbicides (bispyribac-sodium, fenoxaprop + ethoxysulfuron, and penoxsulam + cyhalofop) at four-, six-, and eight-leaf stages.

Weed control treatments	Rate (g ha^−1^)	Root-shoot ratio		
		Four-leaf	Six-leaf	Eight-leaf
*IR population*				
Nontreated control	0	0.66	2.84	0.61
Bispyribac-sodium	30	0.06	0.55	0.67
Fenoxaprop + ethoxysulfuron	45	0.00	0.94	0.70
Penoxsulam + cyhalofop	72	0.00	0.40	0.93
LSD_0.05_		0.15	1.10	0.46
*NE population*				
Nontreated control	0	1.00	2.06	1.99
Bispyribac-sodium	30	0.08	0.74	1.18
Fenoxaprop + ethoxysulfuron	45	0.31	0.53	1.77
Penoxsulam + cyhalofop	72	0.26	0.52	1.97
LSD_0.05_		0.19	0.30	0.89

Abbreviations: NS  =  not significant.

These results suggest that the timing of the application of POST herbicides is critical to achieving maximum efficacy. Early herbicide application (4-leaf stage) provided more effective control of both populations of *E. glabrescens*.

The importance of early application of POST herbicides was also observed in a previous study [19], the authors of which stated that the application of all herbicides at 4-leaf stage provided a 89–98% control of *E. crus-galli* and a more than 95% control of *E. colona*.

In our study, the herbicides provided reductions of 85–100% in seedling survival, 41–81% in shoot biomass, and 75–100% in root biomass in both populations when applied at the 4-leaf stage.

Since all herbicides were equally effective against *E. glabrescens*, these can be used in rotation to prevent a possible evolution of resistance to these herbicides when these are continuously used against the weeds. Fenoxaprop + ethoxysulfuron applied at the 6-leaf stage controlled *L. chinensis* and *D. ciliaris*, while penoxsulam + cyhalofop controlled only *L. chinensis* and bispyribac-sodium controlled only *E. colona* [19].

In our study, we observed that at the 6-leaf stage, only fenoxaprop + ethoxysulfuron reduced seedling survival (65–77%) in *E. glabrescens*. This observation suggests that fenoxaprop + ethoxysulfuron can be used in situations in which herbicides cannot be applied at the early stages due to rain or other reasons.

Failure of all treatments to control *E. glabrescens* at the 8-leaf stage could be the result of higher tolerance of herbicides among bigger and older plants [20]. In a similar study, the efficacy of bispyribac-sodium, fenoxaprop + ethoxysulfuron, and penoxsulam + cyhalofop in *E. crus-galli* and *E. colona* decreased when applied at the 8-leaf stage [19].

The results of our study suggest that for a more effective, season-long, and sustainable weed control in direct-seeded rice systems, weed management methods must be used in combination with one another as a strategy. In no-till systems, the use of a stale seedbed combined with the retention of large amounts of residue, shallow flooding at early stage (0–4 DAS), and application of POST herbicide at the 4-leaf stage of the weed can reduce its density. Since *E. glabrescens* is expected to proliferate in continuous no-till systems, there is also a need to rotate tillage systems. Deep cultivation that would bury weeds below 8 cm is thus suggested. Succeeding cultivation should be shallower to prevent previously buried weed seeds from coming back onto the soil surface. In conventional systems, the stale seedbed method followed by the application of PRE herbicides at low dose followed by flooding at shallow depths may also be an option in controlling *E. glabrescens*. In the absence of PRE herbicides, POST herbicides can be applied at the early stages.

## References

[1] Chauhan BS (2012) Weed ecology and weed management strategies for dry-seeded rice in Asia. Weed Technol 26: 1–13.

[2] Pandey S, Velasco L (2005) Trends in crop establishment methods in Asia and research issues, in *Rice Is Life: Scientific Perspectives for the 21st Century*, K Toriyama, Heong KL, Hardy B, Editors. Los Baños, Philippines: International Rice Research Institute and Tsukuba, Japan: Japan International Research Center for Agricultural Sciences, p 178–181.

[3] Bureau of Agricultural Statistics (2011) *Monthly Agricultural Situation Report* 2011. Available: http://www.bas.gov.ph/?ids=masr&id=20&mon=2011-04. Accessed 2013 Nov 15.

[4] Erguiza A, Duff B, Khan C (1990) Choice of rice crop establishment technique: transplanting vs wet seeding, in IRRI Reserach Paper Series 139. 10 pp.

[5] Abilay WP, Garcia FV, Alcantara JM, De Datta SK (1984) *Changes in input use and grain yields in lowland rice farms in three Philippine provinces*, in *IRRI Research Paper Series No. 100*. International Rice Research Institute: Los Baños, Philippines.

[6] Mandac AM, Kalirajan KP, Flinn JC (1982) Economic limitations to increasing shallow rainfed rice productivity in Bicol, Philippines, in IRRI Research Paper Series 80. 21 pp.

[7] Chauhan BS, Abugho SB (2012) Threelobe morningglory (*Ipomoea triloba*) germination and response to herbicides. Weed Sci 60: 199–204.

[8] Dorji S, Chauhan BS, Baltazar AM, Johnson D (2013) Effect of flooding depth and pretilachlor rate on emergence and growth of three rice weeds: junglerice (*Echinochloa colona*), smallflower umbrella sedge (*Cyperus difformis*), and ludwigia (*Ludwigia hyssopifolia*). Can J Plant Prot 1: 43–48.

[9] Holm LG, Plucknett DL, Pancho JV, Herberger JP (1991) *The World*'*s Worst Weeds: Distribution and Biology*. Malabar, Florida: The University Press of Hawaii. 609 p.

[10] Chauhan BS, Johnson DE (2009) Seed germination ecology of junglerice (*Echinochloa colona*): a major weed of rice. Weed Sci 57: 235–240.

[11] Chauhan BS, Johnson DE (2011) Ecological studies on *Echinochloa crus-galli* and the implications for weed management in direct-seeded rice. Crop Prot 30: 1385–1391.

[12] Diop AM, Moody K (1984) Effect of seeding depth and flooding regime on germination and growth of *Echinochloa glabrescens* . Phil J Weed Sci 11: 65–69.

[13] Rao AN, Moody K (1987) Weed seedlings transplanted with rice seedlings reduce grain yield. Int Rice Res Newsletter 12: 51.

[14] Rao AN, Moody K (1992) Competition between *Echinochloa glabrescens* and rice (*Oryza sativa*). Trop Pest Manage 38: 25–29.

[15] Chauhan BS, Johnson DE (2010) The role of seed ecology in improving weed management strategies in the tropics. Adv Agron 105: 221–262.

[16] Chauhan BS, Johnson DE (2008) Germination ecology of Chinese sprangletop (*Leptochloa chinensis*) in the Philippines. Weed Sci 56: 820–825.

[17] Lee J, Chauhan BS, Johnson DE (2011) Germination of fresh horse purslane (*Trianthema portulacastrum*) seeds in response to different environmental factors. Weed Sci 59: 495–499.

[18] Bolfrey-Arku GE-K, Chauhan BS, Johnson DE (2011) Seed germination ecology of itchgrass (*Rottboellia cochinchinensis*). Weed Sci 59: 182–187.

[19] Chauhan BS, Abugho SB (2012) Effect of growth stage on the efficacy of postemergence herbicides on four weed species of direct-seeded rice. Scientific World J, Article ID 123071, 7 pages. 10.1100/2012/123071 PMC3349310 22619576

[20] Singh S, Singh M (2004) Effect of growth stage on trifloxysulfuron and glyphosate efficacy in twelve weed species of citrus groves. Weed Technol 18: 1031–1036.

[21] Michel BE (1983) Evaluation of the water potentials of solutions of polyethylene glycol 8000 both in the absence and presence of other solutes. Plant Physiol 72: 66–70. 16662983 10.1104/pp.72.1.66 PMC1066170

[22] Chauhan BS, Johnson DE (2008) Influence of environmental factors on seed germination and seedling emergence of eclipta (*Eclipta prostrata*) in a tropical environment. Weed Sci 56: 383–388.

[23] Chauhan BS, Johnson DE (2008) Germination ecology of goosegrass (*Eleusine indica*): an important grass weed of rainfed rice. Weed Sci 56: 699–706.

[24] Eslami SV (2011) Comparative Germination and emergence ecology of two populations of common lambsquarters (*Chenopodium album*) from Iran and Denmark. Weed Sci 59: 90–97.

[25] Schütz W, Milberg P, Lamont BB (2002) Seed dormancy, after-ripening and light requirements of four annual Asteracea in south-western Australia. Ann Bot 90: 707–714. 12451026 10.1093/aob/mcf250 PMC4240361

[26] Chauhan BS, Johnson DE (2009) Influence of tillage systems on weed seedling emergence pattern in rainfed rice. Soil Till Res 106: 15–21.

[27] Boyd NS, Van Acker RC (2004) Seed germination of common weed species as affected by oxygen concentration, light, and osmotic potential. Weed Sci 52: 589–596.

[28] Benvenuti S, Macchia M (1995) Hypoxia effect on buried weed seed germination. Weed Res 35: 343–351.

[29] Egley GH (1986) Stimulation of weed seed germination in soil. *Reviews in* Weed Sci 2: 67–89.

[30] Baskin CC, Baskin JM (1998) Seeds: Ecology, Biogeography, and Evolution of Dormancy and Germination. San Diego, CA: Academic. 666 p.

[31] Chauhan BS, Migo T, Westerman PR, Johnson DE (2010) Post-dispersal predation of weed seeds in rice fields. Weed Res 50: 553–560.

[32] Crutchfield DA, Wicks GA, Burnside OC (1985) Effect of winter wheat (*Triticum aestivum*) straw mulch level on weed control. Weed Sci 34: 110–114.

[33] Facelli JM, Pickett STA (1991) Plant litter: its dynamics and effects on plant community structure. Bot Rev 57: 1–32.

[34] Gadde B, Bonnet S, Menke C, Garivait S (2009) Air pollutant emissions from rice straw open field burning in India, Thailand and the Philippines. Environ Pollution 157: 1554–1558. 10.1016/j.envpol.2009.01.004 19201513

[35] Chauhan BS, Johnson DE (2009) *Ludwigia hyssopifolia* emergence and growth as affected by light, burial depth and water management. Crop Prot 28: 887–890.

[36] Kent RJ, Johnson DE (2001) Influence of flood depth and duration on growth of lowland rice weeds, Cote d'Ivoire. Crop Prot 20: 691–694.

[37] Pons TL (1982) Factors affecting weed seed germination and seedling growth in lowland rice in Indonesia. Weed Res 22: 155–161.

[38] Smith RJJ, Fox WT (1973) Soil water and growth of rice and weeds. Weed Sci 21: 61–63.

[39] Tuong TP, Bouman BAM, Mortimer M (2005) More rice, less water: integrated approaches for increasing water productivity in irrigated rice-based systems in Asia. Plant Prod Sci 8: 231–241.

[40] Pablico PP, Moody K (1993) Effect of flooding duration on the performance of pretilachlor + fenclorim in wet-seeded and transplanted rice. Phil J Weed Sci 20: 56–61.

[41] Chauhan BS, Prabhjyot-Kaur, Mahajan G, Randhawa RK, Singh H, et al (2014) Global warming and its possible impact on agriculture in India. Adv Agron 123: 65–121.

